# Supplementary material for: Myeloid Ecotropic Viral Integration Site 1 Promotes Vascular Smooth Muscle Differentiation through Activation of Myocardin
Source: bioRxiv. 2026 Sep 28:2026.09.26.751066. Preprint. [Version 1] doi: 10.64898/2026.09.26.751066 (PMC13648518; doi:10.64898/2026.09.26.751066)

(Wally et al, supplmentary Fig 1)

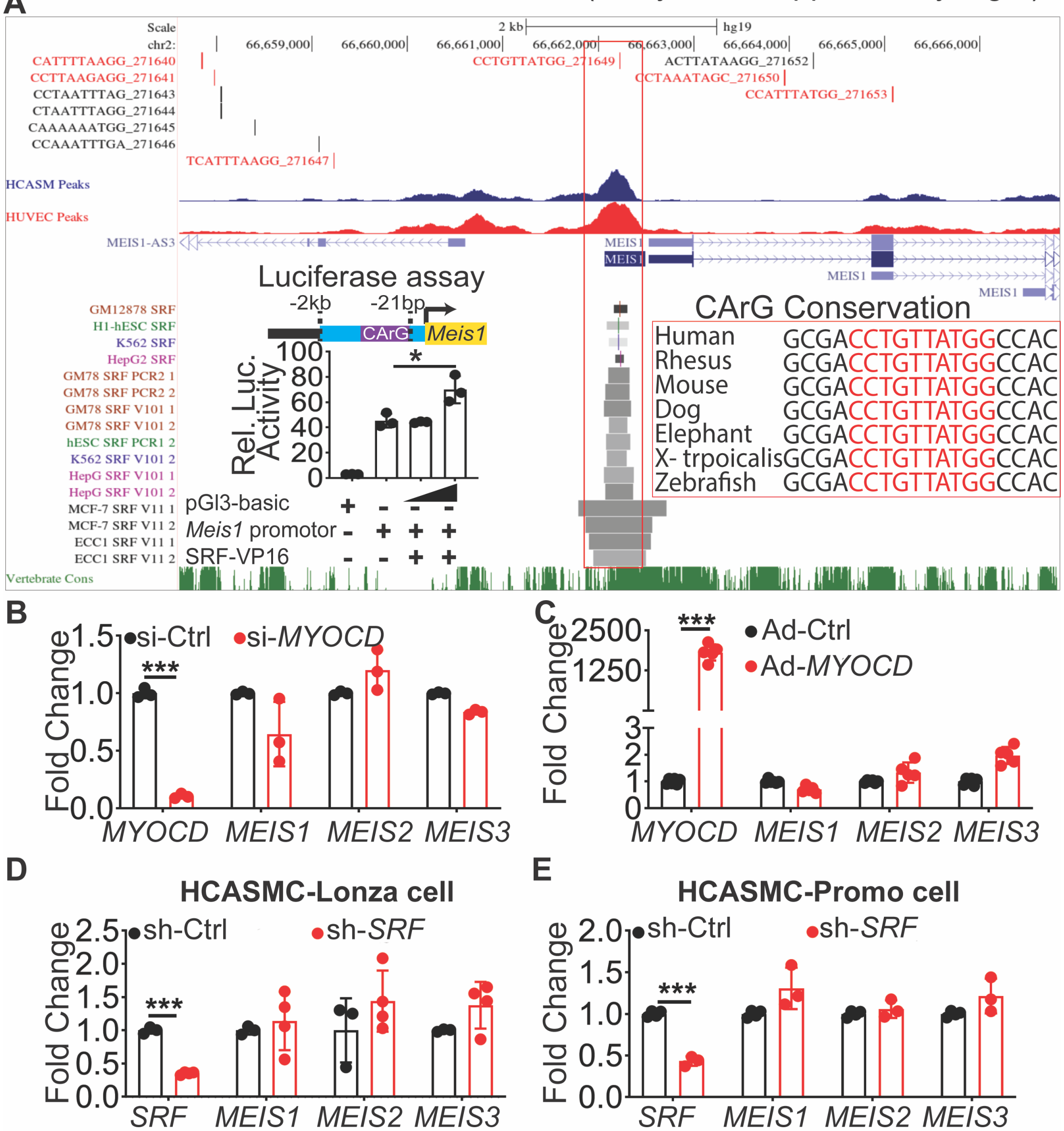

(Wally et al, supplmentary Fig2)

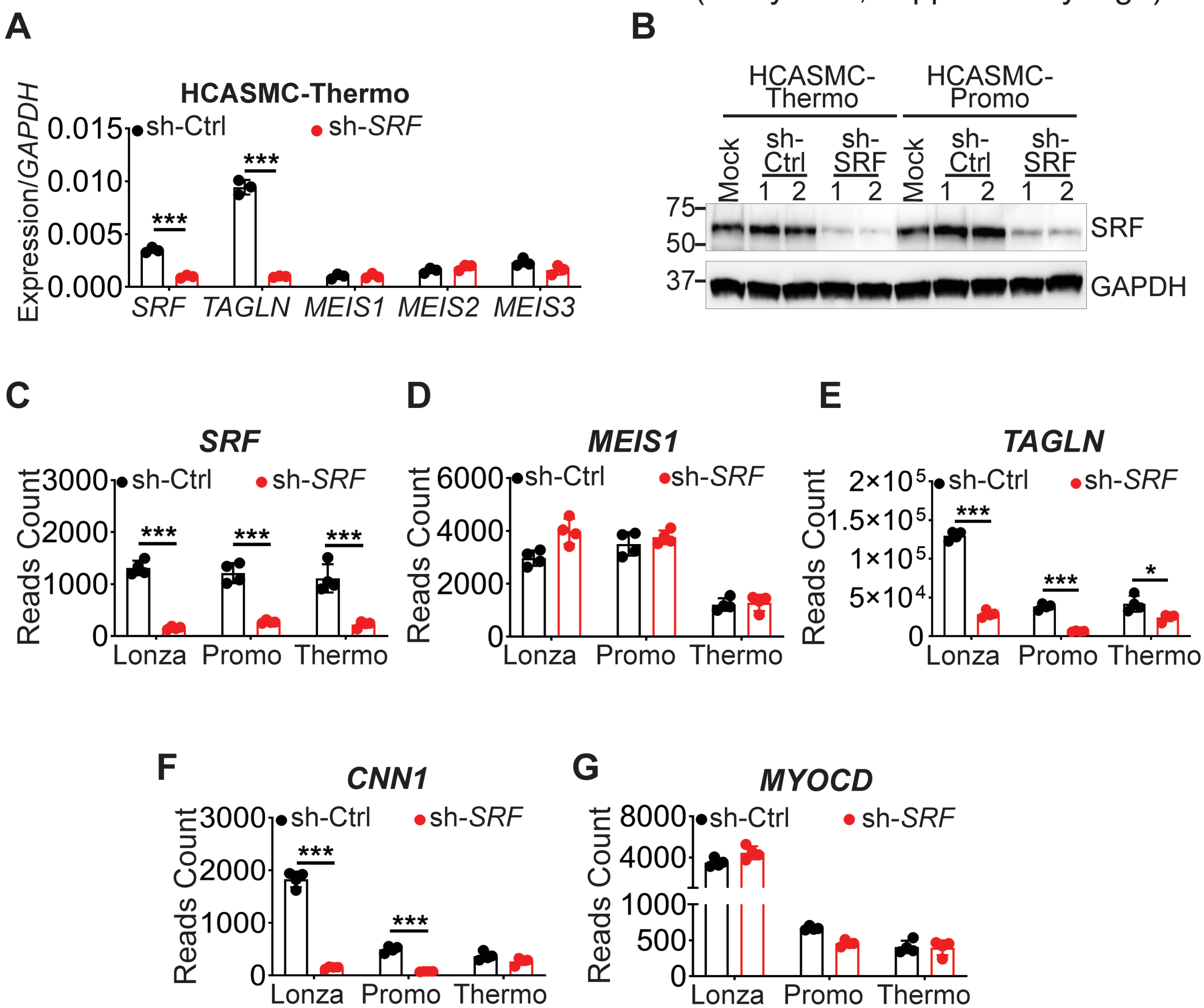

(Wally et al, supplmentary Fig3)

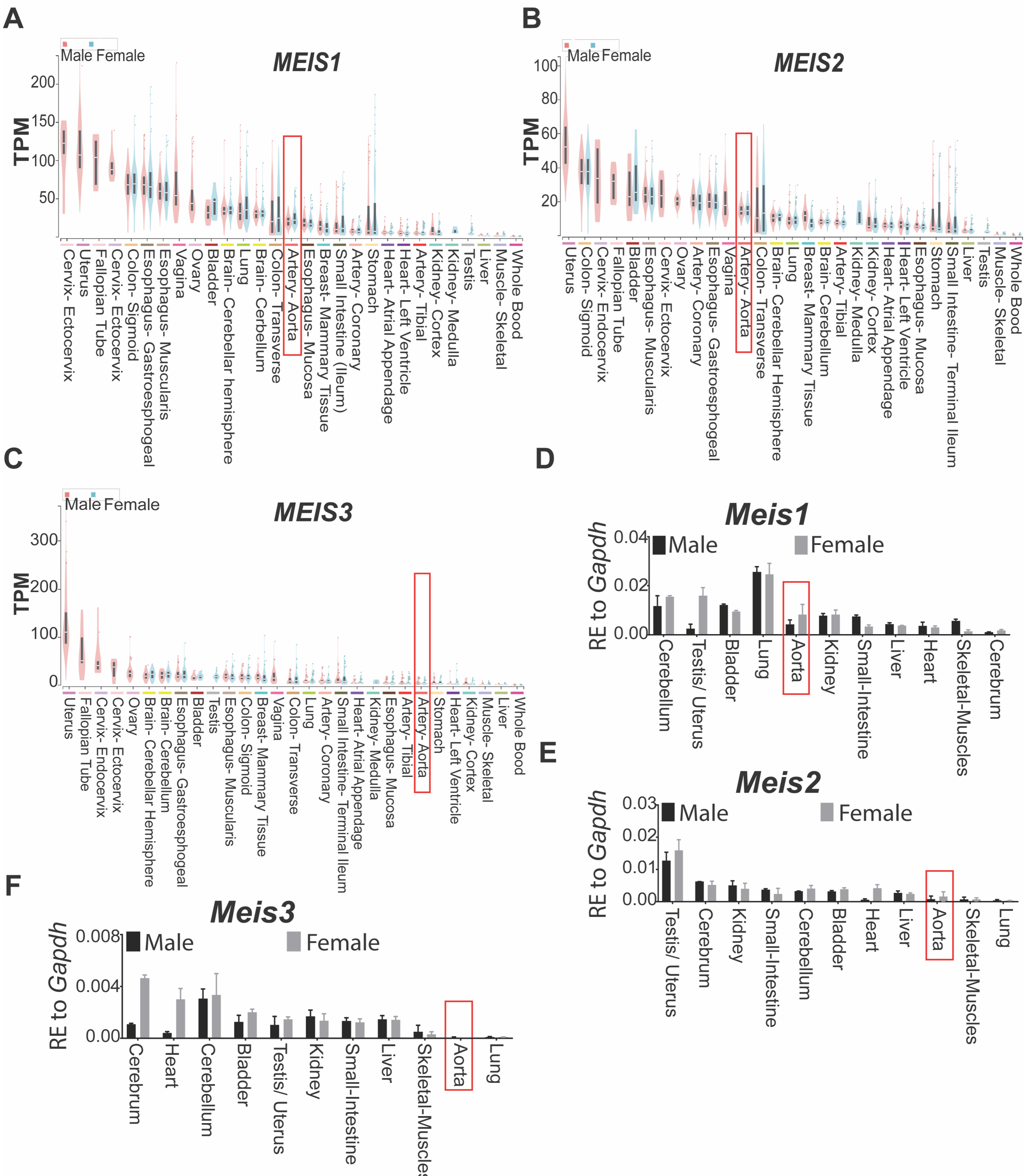

(Wally et al, supplmentary Fig4)

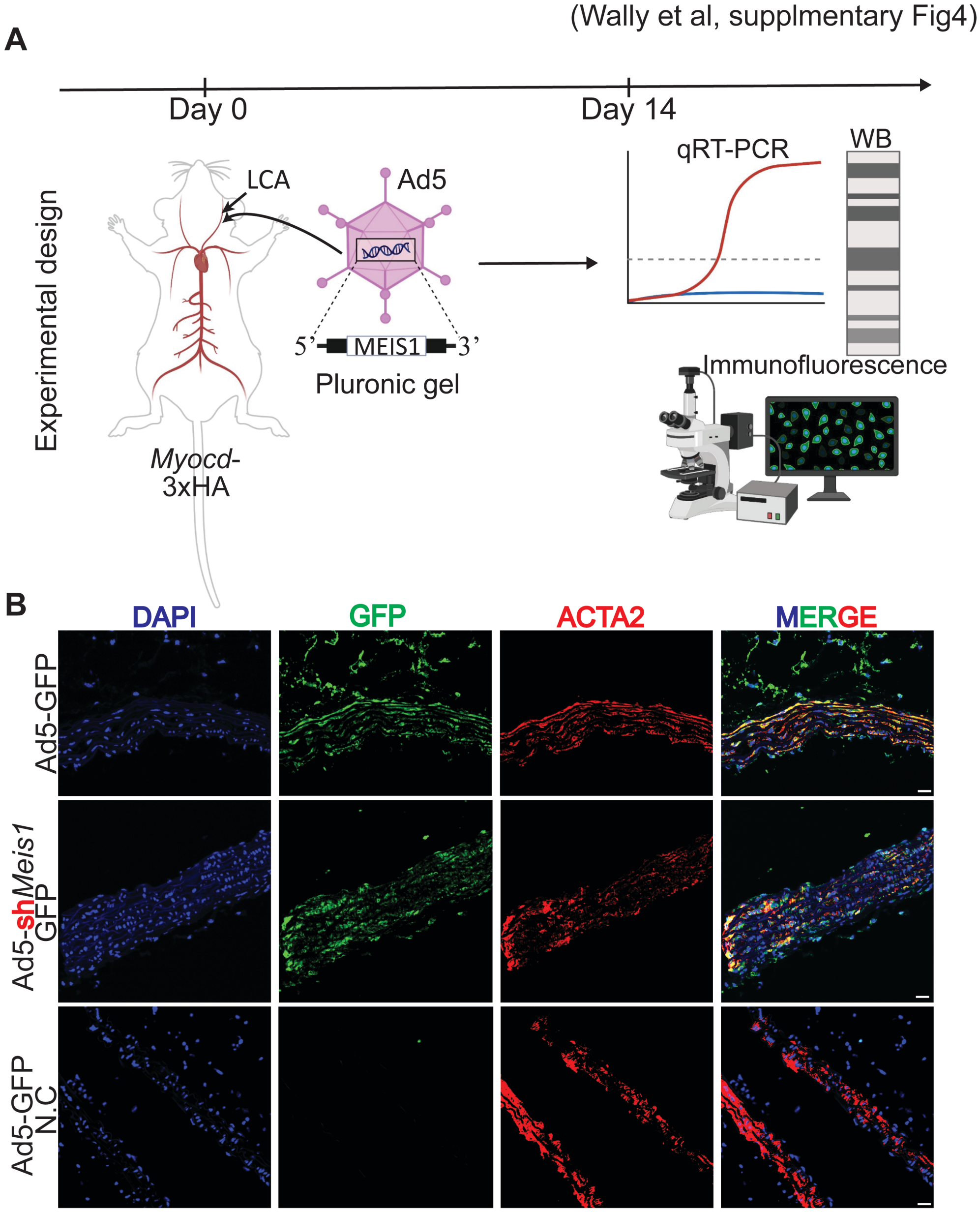

(Wally et al, supplmentary Fig5)

A

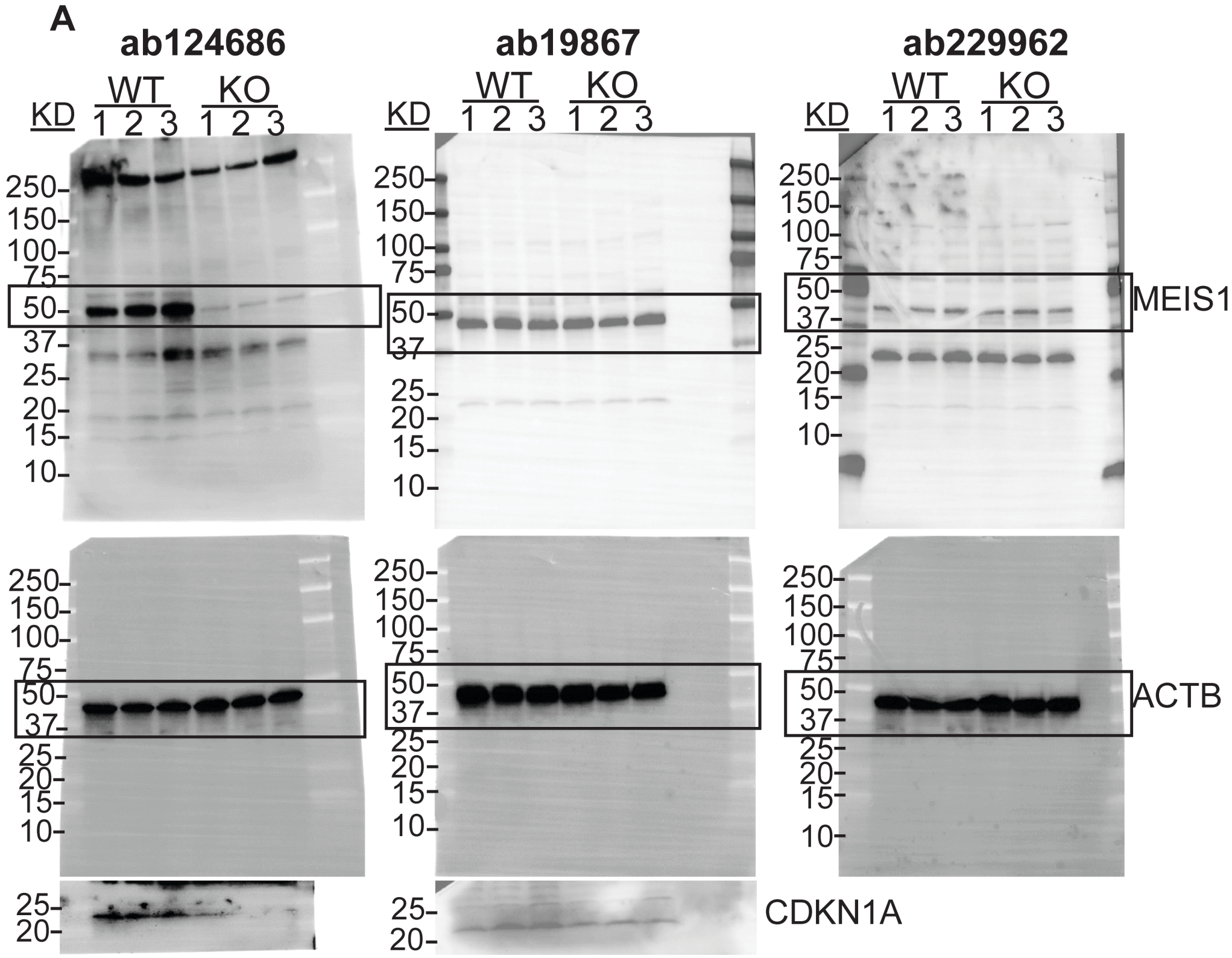

B

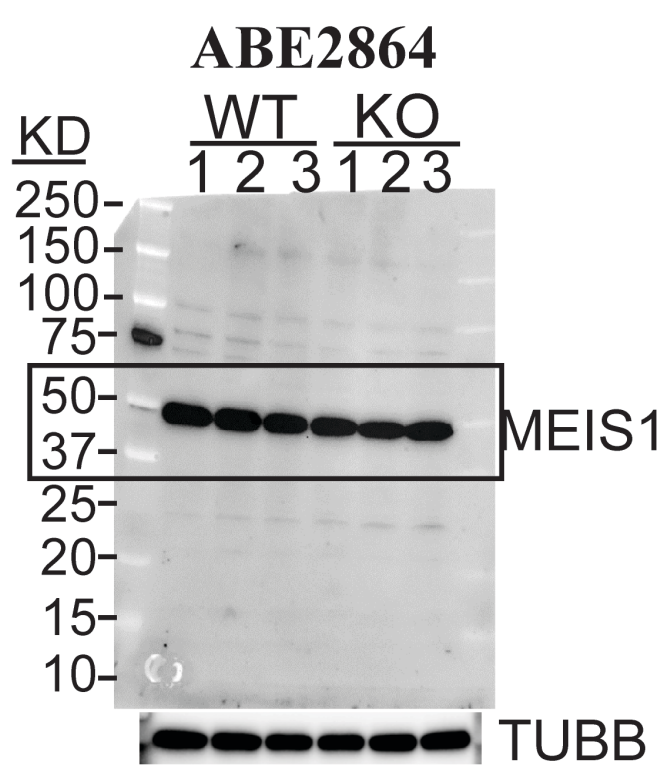

Supplement: Supplementary file 1 [file media-1.pdf]
